# The Opium Curse and a Prevention*Read at the late meeting of the Tennessee Medical Society.

**Published:** 1895-07

**Authors:** T. J. Happel

**Affiliations:** Trenton, Tenn.


### THE OPIUM CURSE AND A PREVENTION.*

BY T. J. HAPPEL, A.M., M.D.,

TRENTON, TENN.

In a paper written a few years ago, entitled "Morphinism in its Relation to the Sexual Functions and Appetite, and its Effects on the Offspring of the Users of the Drug," I gave a detailed account of eight families, where the mother was addicted to the use of morphine. Since that paper was completed and read, I have had occasion to note the effect of the drug in a few other families, and the results there have served to confirm the views set forth in that paper, viz.: that the children of mothers who are habitual users of the drug, in the majority of cases die within a week of their birth cyanosed, from an incomplete development of the heart; second, that if they survive the first year they are puny, delicate, nervous children, lacking in everything going to make up a well equipped boy or girl, mentally and physically; third, should any of the offsprings of such mothers attain to adult life, they become either morphine habitués or drunkards. That the effect upon the mother is such as to transmit to her offspring the disease as a heredity, just as is tuberculosis; not that the disease itself is transmitted, but that "a condition, a soil, a nidus, or whatever you may please to call it, is handed down to the child, and some fortuitous circumstance develops the disease."

If this is a true picture, and I am constrained to believe that it is, a great responsibility rests upon some one to proclaim the evil everywhere and enforce every possible means of preventing it. The most important branch of medicine to-day is that of prophylaxis. Disease is easier to prevent than to cure. To cure this curse, the using of the drug must be prevented. Once in its toils, to abandon the use of it is one of the greatest undertakings. It has been truly and aptly said that to abandon its use is to suffer the tortures of the damned. Further, without the full and free consent of the patient and his or her hearty coöperation, "to break the morphine habit" is well-nigh impossible. I would say

*Read at the late meeting of the Tennessee Medical Society.

nothing to discourage any one, but my own observation teaches me that it is far easier to abandon alcohol than opium. The using of opium is increasing daily. Public sentiment cries out more loudly to-day than ever before against the use of alcoholic drinks. Public opinion is against it. The dram-drinker is not countenanced in polite society. All business enterprises and corporations are discriminating against those who use alcohol. The great railroad systems put as a first question to an applicant for a position in their employ, "Do you use alcoholic drinks of any kind; if so, how much and what?" Not content with the answers of the applicant, his character upon that point is clearly investigated at home where he is known. Should it appear that he uses intoxicants, he is at once refused a position. Further, an employee is at once dismissed if found at all under the influence of liquor. One drink is recognized as, to that extent, unfitting a man for a trustworthy position in the employ of the company.

One of the most important questions in all life insurance applications is, "Does the applicant use any stimulant in the form of any alcoholic drink?" Should it be shown that he is a regular drinker, he is at once rejected. The fraternal orders are all blacklisting saloon-keepers and bartenders.

These things are cited to show the current of public opinion. The retail sale of liquor is regarded by communities as disreputable; but are we not "straining at a gnat and swallowing a camel"? Forty years ago the United States imported 72,000 pounds of opium; in 1880, 372,000 pounds, and in 1893, nearly one million pounds. This increase is largely out of proportion to the increase of population and the legitimate demands of medicine. The medical and surgical world were never more united upon any one question than in an effort to discourage the use of opiates in many diseases where it was formerly thought and taught to be a *sine qua non*.

Within the twenty years of my practice I can see that not more than one-fourth as much morphine is used now as was used when I began work. The teaching of to-day is, that when in doubt as to the propriety of an opiate, do not give it.

The medical profession, then, is not to be censured for the increase in the consumption of morphine. Its use is continually discouraged by all reputable practitioners of medicine.

Of course, in incurable diseases, in their last stages, such as cancer and such like, a sufferer is excusable if he uses the drug, but not otherwise. The physician is frequently unjustly blamed because Mrs. A or B, or Mr. C or D, uses an opiate. In not one case in a thousand of this kind should any blame attach to the physician; because had he or she never taken a dose of an opiate, except when it was prescribed by the physician, there would have been no trouble; but having once found relief, concludes that he can free himself from pain, and at the same time save the cost of calling the physician, by bringing out the morphine bottle or the opium pill that had been put by for an emergency. Relief once obtained in this way invites another trial, and soon the habit becomes fixed and the party becomes a morphine habitué, or an opium-eater or smoker.

Is the physician to blame in such a case? Nay, verily; he did right. His patient must shoulder the blame. The severity of the pain when the physician was called demanded relief; and a relaxation of the spasm of the muscles was necessary before the cause of his suffering could be removed. For this purpose the opiate was given. The physician had nothing to do with the subsequent doses. Had they not been sold to the patient he would not have been tempted and would not have fallen. A dose of an opiate, excepting paregoric or laudanum, should never be administered except as ordered by a physician.

On September 13, 1894, this fact was most forcibly impressed upon my mind. I was called to see a two-year old child suffering from enteritis. The patient had been well treated by the attending physician with no improvement. On consulting in regard to the case, a course of treatment was readily agreed upon, and it was remarked by the attending physician that he had not been able to quiet the child with what he considered full doses of an opiate, repeated every two or three hours. The mother, when called and questioned in regard to the dose of paregoric given to the child, stated that she was administering a full half-teaspoonful every two hours, but that it had no effect upon the child.

To an inquiry as to whether she had ever given the child any form of an opiate, she answered that she occasionally gave it a small dose of morphine; that she began using it for the relief of crying spells when younger. Asked to show how much morphine she gave at a dose, she measured out a full third of a grain, for a child about two years old—fully seven times a dose. This at once revealed why no effect had been obtained from the medicines already administered. This dose of morphine was measured from a bottle produced from the family medicine shelf. No physician had ever advised morphine for the child. Too often is it the case, the first investment made in a family when a child is born is a bottle of "soothing syrup," Bateman's Drops, somebody's "quick cure for colic," or some such like remedy. All of them are sold and warranted to contain no morphine or opium. The various consumption cures and cough remedies, with high sounding titles, —too many, often with the certificate of some business man, lawyer, or preacher appended, attesting their virtues,—put upon the market at the present time, and sold over the druggists' counter, are to blame for many a ruined member of society, cursed with the opium habit. This is not overstating the matter, rather understating it. Ninety-nine out of every hundred of those varied preparations sold contain opium in some form, although they state upon their face that they contain no "morphine." They may not contain simply morphine, but the foundation of them is some preparation of opium, and they sell because they stop the cough and make the "patient feel good." A dose of paregoric, laudanum, opium, or morphine would do the same. Once having obtained relief, that patient advises some one else of the wonderful relief and benefit he or she obtained, and the new victim tries it, and soon a fully fledged opium habitué results. Should a dose of such stuff be given to the offspring of a morphine-using mother, the smouldering fire at once begins to burn. The thing necessary to develop the habit has been given; the match has been applied to the rubbish pile, and it at once blazes up and burns with a flame that cannot be subdued. Doctor Crothers, of Hartford, Conn., cites many cases in proof of this statement. One will answer my purpose: "To parents, both neurotic, and probably opium-users, a child was born. They died, leaving the child nervous and irritable. Morphine was accidentally given to quiet it, and from that time forward it became delirious without a daily dose of morphine. At five years of age it died a confirmed morphine-user." The literature of the day abounds in such cases. Was the physician to blame there? Nay, verily.

I have in my own experience met with a similar case where two sons, born to an opium-eating, morphine-using father, shortly after budding into a promising young-manhood, began the use of whisky and morphine, and soon became wrecks mentally, morally, and physically; one dying not long since; the other being in every sense of the term a wreck. Though living, he is worse than dead. Dr. Jules Rochard, in the *Union Medicale*, draws a gloomy picture of the increase of the morphine habit in France and elsewhere. The habit, he finds, becomes incurable at the end of six months of indulgence. The fair sex and the doctors are, in his opinion, most deeply addicted to the use of morphine. Women seek less to hide the vice than do men. As a rule, men, and especially medical men, take the greatest pains to hide their vice:—hence the number of those who use the drug cannot be correctly estimated.

In many cases parties have used the drug for years without being suspected of such a habit. The current literature abounds in many such cases. A few years ago I was treating a medical friend in a case of pneumonia, and at the time was much worried by the irregular effect obtained from the medicines administered, and at the time suspected that an opiate was being used but could get no positive proof of the matter. A few days since I learned, beyond question, that my quondam patient was a confirmed morphine-user. These are not exceptional cases. Numbers of just such cases, not only among medical men but also among the laity, are cited in Hare's "Practical Therapeutics." This ability to conceal the habit is one of the distinctive points in the use of alcohol and opium.

A native Chinese preacher, in comparing the two vices, stated that he found this one striking difference between the effects of the opium vice among his countrymen, and those produced by alcoholic intemperance among Americans: "When the Chinese opium-smoker comes home at night, he does not abuse his children and kick his wife; his wife kicks him."

The use of opium, in some of its forms, is, in my opinion, on the increase, and whilst the medical profession cannot be blamed therefor, it should, however, be held to some extent responsible for such a state of affairs, because, standing as we do, or should, upon the watch-towers, we do not proclaim boldly to the families under our charge the dangers lurking in the use of all such remedies. We do not make proper inquiries about the progress of our little ones, and keep the parent posted upon the various household remedies administered. Many deaths have been reported in the cases of young children a few months old, from unknown causes, where a careful inquiry would show beyond cavil that they were cases of opium poisoning. I need not pursue this branch of my subject any further. Any one by a little investigation can establish the truth of it. The druggist sells the medicine, which he buys, for the money that is in it. He does not trouble himself about the composition of it. He reads the label, and notes what it claims to cure, and when a human being calls for a remedy to meet a certain ailment, he recalls the fact that he has on his shelves a bottle warranted to cure just such a case. He takes it down, assures his victim of the efficacy of the mixture, and sells it to him, pocketing at the same time a handsome profit. The remedy may do more harm than good, yet the druggist is not responsible. He has the stuff for sale, and the man wanted it and got it. This could be prevented, first, by allowing no patent or proprietary medicine to be sold over the counter which does not show on its label its exact and true composition. Any falsification in such matter should be punishable by a heavy fine. Secondly, no one should be allowed to sell drugs who is not a qualified pharmacist. In other words, the present pharmacy law should be improved and made to apply to the whole State.

Were these ideas carried out and enforced as laws, then all "soothing syrups," "consumption cures," "microbe killers," and such like would show upon their face their exact composition, and the mother would know with what deadly stuff she was drenching her child; would realize how rapidly she was laying the foundation upon which, in time, would develop a fully fledged opium-eater.

In the next place, the law regulating the sale of poisons should be rigidly enforced. The provisions of the law are plain. Morphine, opium, and laudanum are poisonous, and will kill with as much certainty as will strychnine, arsenic, and such like poisons.

Not one druggist in one hundred complies with the law. No register is kept in which to note the sale of poisons. Anything called for is sold, and no questions are asked, except, "Where is the money?" When that is produced the sale is completed, and the drug is delivered. Often and over, a child of less than ten years of age steps into a drug-store with a fifty-cent piece and a small scrap of paper inscribed with one word, "Morphine." No name is signed. No questions are asked. The bottle of morphine is wrapped up and passed to the child over the counter. A death may follow on the sale, but the morphine cannot be traced. No register is kept in which the sale is noted, and hence no proof exists that A or B sold the poison, and no prosecution follows. Druggists say that the amount of opiates sold is beyond the comprehension of the average doctor even; that they sell it day by day without knowing or even inquiring for whom it is bought. Such should not be the case; but so long as any irresponsible party, or child of tender years, can buy without let or hindrance, the awful traffic will grow. If the laws were enforced to the letter, and the name of the purchaser of every grain of morphine entered in a public ledger, open to the inspection of friend or foe, where the buyer did not present the prescription of a reputable physician for the drug, a halt would promptly be called.

Even a chronic opium-user would draw back and hesitate to allow the public to know the quantity of the drug he or she consumes, for it is an incontrovertible fact that open and notorious morphine habitués will always minimize the amount of the drug used. Time and again, in answer to the question as to how much the individual used, have I been given a quantity of about one-third or one-fourth of the amount that I knew was consumed by them. The opium-eater does not want the public to know how much he consumes. The habit is carried on in secret for years, till it bursts on the public in some unexpected way.

The user of the drug will sacrifice money, place, position, honor, nay, verily, in some cases even virtue itself, to obtain the vile stuff, and until some sacrifice of this kind is made upon the altar of appetite the habit remains concealed. It is a habit more seductive than alcohol, because, so to say, for a long time it can be kept as a private vice, whilst the habit of using alcoholic drinks cannot be kept long concealed. The odor upon the breath, the bloom upon the nose, the flushed face, the protruding abdomen, and many other signs soon proclaim the dram-drinker, whilst opium can long be indulged in without being generally known. The sleepy state, as the drug wears off, can be accounted for by a rest broken by watching a sick friend, or an undue nervousness from some cause.

The female sex does not often, in the country and small towns, become addicted to the use of alcohol, whilst opium claims more of them as its devotees than of the male sex. The latter attracts men and women—the former, as a rule, men only.

If the vicious habit—the use of opium in any of its forms—imposed a penalty only upon the person using, then the damage done would not be so great, but the unborn child suffers—the evil is entailed. The sins of the mother are visited upon the children of even the third and fourth generations.

The child, through no fault of its own, must suffer from the evil habits of one or both of its parents. This state of affairs should not be. The strong arm of the law, which looks closely after the moneyed interests of the child, left an orphan, should be invoked to prevent a far worse condition than that of a moneyless orphanage. The child, with no constitution at all, or with one leaning to vice and immorality, is a proper charge upon the State. If the State does not engage in prophylaxis, later on she will find that curative measures are both costly and, in many cases, without any favorable results. Let, then, the physician do his part, and the laity their part, and let both see that the letter of the existing laws is carried out, and both work together to get better laws enacted, and a great step will be taken in the fight against opium. Prevention is better and more available in this instance than any other course.

As both a preventive and curative measure, all chronic inebriates and opium-eaters should be committed to the insane asylums for treatment, and kept there till completely cured. No half-way measures should be taken in such cases. Laws should be passed broad enough in their scope to reach all such cases, and so drawn as to meet all objections that could possibly be urged against them upon the personal liberty question. They should, to all intents and purposes, be treated as persons dangerous to the public, and not permitted to be at large.

The insane dodge is put up by them when they violate the laws of our State, and this method of treatment simply would recognize the possibility of the existence of such a state of affairs.

This paper has been written to air no pet theory of my own, but to invite the attention of the society to a great and growing evil.

1. Make our druggists obey the laws. Protect ourselves against blame.

2. Let the patients know that if they will use the drug, the unborn generation shall be protected—so far as can be done—by putting the users where the drug cannot be gotten and the habit is broken.

